# The motion of catalytically active colloids approaching a surface†

**DOI:** 10.1039/d4sm01387e

**Published:** 2025-03-05

**Authors:** Julio Melio, Solenn Riedel, Ali Azadbakht, Silvana A. Caipa Cure, Tom M.J. Evers, Mehrad Babaei, Alireza Mashaghi, Joost de Graaf, Daniela J. Kraft

**Affiliations:** a Huygens-Kamerlingh Onnes Laboratory, Leiden University P.O. Box 9504 2300 RA Leiden The Netherlands kraft@physics.leidenuniv.nl; b Medical Systems Biophysics and Bioengineering, Leiden Academic Centre for Drug Research, Faculty of Science, Leiden University 2333CC Leiden The Netherlands; c Institute for Theoretical Physics, Center for Extreme Matter and Emergent Phenomena, Utrecht University Princetonplein 5 3584 CC Utrecht The Netherlands

## Abstract

Catalytic microswimmers typically swim close to walls due to hydrodynamic and/or phoretic effects. The walls in turn are known to affect their propulsion, making it difficult to single out the contributions that stem from particle-based catalytic propulsion only, thereby preventing an understanding of the propulsion mechanism. Here, we use acoustic tweezers to lift catalytically active Janus spheres away from the wall to study their motion in bulk and when approaching a wall. Mean-squared displacement analysis shows that diffusion constants at different heights match with Faxén's prediction for the near-wall hydrodynamic mobility. Both particles close to a substrate and in bulk show a decrease in velocity with increasing salt concentration, suggesting that the dominant factor for the decrease in speed is a reduction of the swimmer-based propulsion. The velocity-height profile follows a hydrodynamic scaling relation as well, implying a coupling between the wall and the swimming speed. The observed speed reduction upon addition of salt matches expectations from a electrokinetic theory, except for experiments in 0.1 wt% H_2_O_2_ in bulk, which could indicate contributions from a different propulsion mechanism. Our results help with the understanding of ionic effects on microswimmers in 3D and point to a coupling between the wall and the particle that affects its self-propulsion speed.

## Abstract 2

Catalytic microswimmers are typically studied close to walls, where they are known to slow down upon addition of salt. By lifting them up, we showed how their speeds change with distance from the wall, with and without salt.

## Graphical abstract

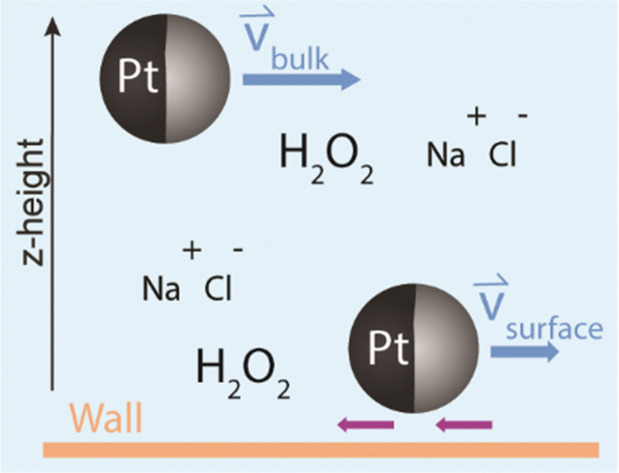

Catalytic synthetic microswimmers^1–3^ are great model systems for living active agents like motile bacteria,^4–6^ algae,^7^ and sperms.^8^ When dispersed in a fuel solution these colloidal particles self-propel as a result of solute gradients generated by the asymmetric catalytic decomposition of the fuel on the swimmer's surface.^9–15^ In experiments, synthetic active particles typically have affinity for surfaces, resulting in them moving close to substrates. The motility of their biological counterparts close to boundaries, however, is very different from bulk motility.^4,8,16–19^ Similarly, also for synthetic swimmers the properties of a nearby wall were shown to have a significant impact on *e.g.* the propulsion speed of the particle^20–23^ and the swimmer-wall separation,^24^ suggesting the presence of a hydrodynamic and/or phoretic coupling with the wall. Additionally, previous work from our group has suggested that the interaction of the wall with the chemical species generated by the catalytic microswimmer can lead to the occurrence of counter flows near the substrate that could affect the net velocity of the swimmer.^20,24^

More clues about potential wall–particle interactions stem from experiments with salt. The speed of swimmers near substrates has been shown to drastically reduce upon the addition of sodium chloride or other charged species.^11,12,24^ This observation provides interesting insights into the nature of the propulsion mechanism of active colloids. The mechanism itself remains under discussion and might have contributions from momentum transfer, as well as neutral and/or ionic self-diffusiophoresis and electrophoresis.^12,20,24–26^ The near-substrate observations of ion induced slowing down suggests an electrophoresis-based propulsion mechanism,^12,25^ or could result from a reduction of ionic near-wall counter flows.^24^ The latter proposal would reconcile observations of a lack of speed variation with the zeta potential of the bare particles, as well as those of a constant swimming height from the substrate at various salt concentrations.^24^ This would make the presence of a wall crucial to observe this salt-induced slowing down, and conversely suggest that the speed of the same particles in bulk, far away from the wall, would be unaffected by the addition of salt.

Motivated by these different proposals, we here compare speeds in bulk and close to a substrate to isolate a possible contribution from the osmotic flows generated on the substrate (*cf.*Fig. 1a). To do so, we performed measurements on active particles that were lifted into the bulk solution with the help of acoustic tweezers, see Fig. 1b, and compared their behavior at different salt concentrations. Bulk experiments were performed at heights ranging from 10 to 30 μm above the substrate, which is greater than, and at the smallest separations comparable to, the decay length of the phoretic flows around the particle.^27^ We observed a decrease in velocity with increasing ionic concentration which is similar for particles in bulk and close to the substrate. This suggests that adding ions primarily affects the particle-based contributions to the propulsion. If a significant osmotic flow along the wall would be present and affect the motion of the active particles, then it only appears to have a minor effect on the self-propulsion speed.

**Fig. 1:**
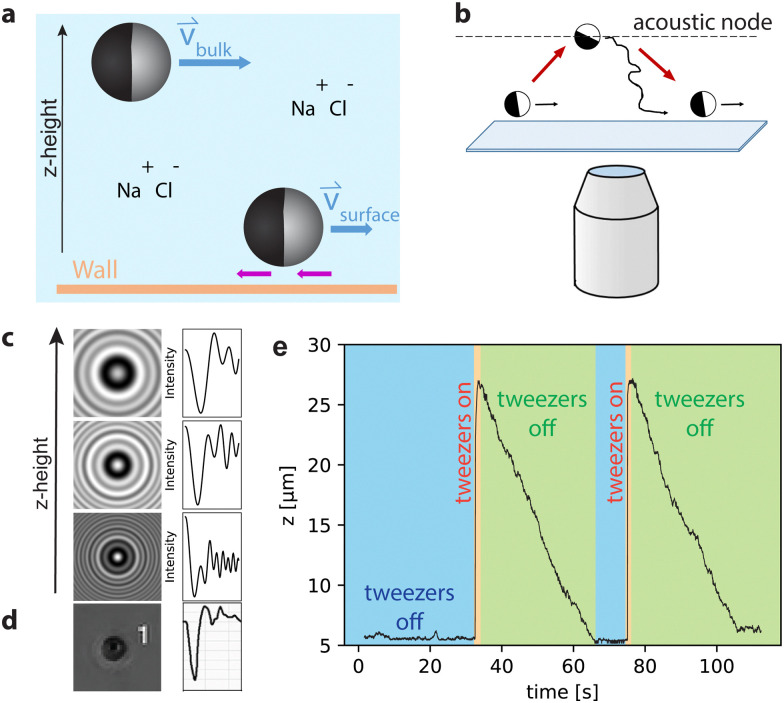
Acoustic tweezer experiments. (a) Close to the substrate, active particles significantly slow down upon the addition of salt. By observing self-propelled particles in bulk, we can determine whether these salt effects are wall-effects or if salt also affects the bulk behavior. The purple arrows represent the counter flows that occur near the wall. (b) Schematic drawing of the acoustic tweezers setup with acoustic node. (c) Three exemplary predictions of scattering patterns for a polystyrene particle at different *z*-heights with the corresponding radial intensity profile. This information is needed to connect the holographic signal to the distance from the focal plane. (d) Experimental scattering pattern snapshot with the radial intensity profile for a particle ≈10 μm above the substrate. (e) *z*-Coordinate trajectory for a passive particle that is lifted up with the acoustic tweezers and sediments twice.

## Experimental

### Experimental setup

In all experiments, we used (4.50 ± 0.14) μm sized polystyrene (PS) particles half-coated with a 5 nm thick Pt/Pd (80/20) layer.§§The metal layer is applied on one side, leading to a maximum radial thickness of 5 nm at a pole that gradually decreases to 0 at the equator. These were rendered active by dispersing them in a 0.1 wt% or 0.5 wt% aqueous hydrogen peroxide (H_2_O_2_) solution. In this system of catalytic synthetic microswimmers, self-propulsion is driven by solute gradients generated through the catalytic decomposition of H_2_O_2_ at the Pt/Pd cap.^1,11^ The active particles were lifted into the bulk using acoustic tweezers paired with holographic microscopy. The measurements were performed in a G2 AFS microfluidic chip holder using an AFS-G2 acoustic tweezers setup from Lumicks B.V. with a motorized *z*-stage mounted on an inverted microscope with a Nikon 20× air objective. For bulk measurements, the particles were lifted up to the acoustic node ±20 μm above the substrate using a standing acoustic wave. When the acoustic field is switched off, the particles were free to self-propel in 3D and the particle positions were recorded in all three dimensions.

### Extraction of particle position

To obtain a particle's *z*-coordinate, a look-up-table (LUT) with a step-size of 100 nm was produced for each particle in the field of view before generating the acoustic wave (Fig. 1d). The LUT comprises the scattering patterns of the particle at specific distances from the focal plane. This allows the radial scattering patterns recorded for that same active particle far from the substrate to be translated to a particle height. The presence of the metal cap influences these radial scattering patterns based on the particle orientation. However, we neglect this effect and treat our particles as isotropic in terms of their radial scattering pattern. We use the small cap thickness and the fact that an individual LUT is recorded for each particle before it is lifted to justify this approximation. While this does not provide us with information on their orientation, we still obtain a good measurement of the particle height. For illustration purposes, examples of scattering patterns of PS-particles at different *z*-heights and the corresponding radial intensity profiles, which were calculated using the python package Holopy, are shown in Fig. 1c. The *x*-, *y*-, and *z*-coordinates are obtained by particle tracking methods using the LabVIEW software provided by Lumicks B.V. Fig. 1e shows a typical trajectory of an experiment with a passive particle in water. After sedimentation in the microfluidic cell, the particle first is close to the substrate (blue section). Upon switching on the acoustic tweezers, the particle is lifted to the acoustic node (orange section), from where it sediments back to the bottom of the cell when the tweezers are switched off (green section). Active particles were observed to move both downwards as well as upwards in the experimental cell after being released from the acoustic trap.^28^ 2D experiments close to the substrate were performed after sedimentation of the particles whilst the acoustic tweezers were off. Experiments on the substrate were complemented by additional measurements using an inverted microscope (Nikon Ti-E) equipped with a 60× water immersion objective (NA = 1.2). The particle motion was imaged at a framerate of 20 fps.

## Results and discussion

### Salt addition experiments

**Fig. 2:**
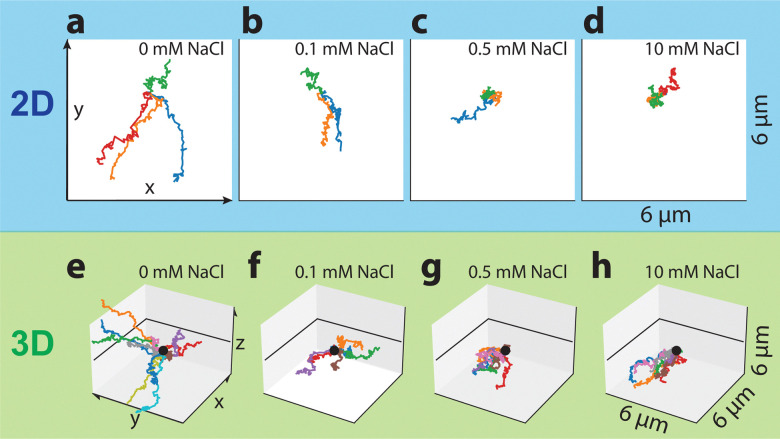
Influence of salt on the activity of catalytic microswimmers in both 2D and 3D. Trajectories of active particles for 2D experiments (a)–(d) and 3D experiments (e)–(h). All trajectories are plotted for 100 consecutive frames (with a frame rate of 18.9 fps corresponding to about 30 s) and have been moved to start in the same point.

To compare the effect of salt on the particle motion in bulk and close to the substrate, we performed a series of experiments at different NaCl concentrations (*c*_NaCl_). 2D and 3D measurements, with the acoustic tweezers off or on, respectively, were taken at *c*_NaCl_ = 0, 0.1, 0.5, and 10 mM in an aqueous H_2_O_2_ solution with a concentration of 0.1 wt% or 0.5 wt%. Exemplary associated 30 s trajectories for increasing salt concentrations are shown in Fig. 2, where different colors indicate different particle trajectories. Panels a–d correspond to colloids self-propelling at the bottom of the measurement cell and thus close to a wall and hence are shown as 2D plots. Panels e–h correspond to colloids moving in 3D far away from the substrate and are shown as a 3D plot. In both cases, the length of the trajectories clearly decreases with increasing salt concentration demonstrating that the propulsion speed of the particles in bulk and close to the substrate is strongly dependent on the presence of salt.

### 2D MSD analysis

Next, we make this assessment quantitative by extracting the effective diffusion coefficients *D* and velocities *v* from the particle trajectories by calculating and fitting the mean-squared displacement (MSD). In experiments close to the substrate, the particles are expected to have a fixed orientation with respect to the substrate.^29,30^*D* and *v* are then obtained by fitting the experimentally measured MSDs with

1 〈*r*^2^〉_2D_ = 4*Dτ* + *v*^2^*τ*^2^,

which captures the behavior in 2D^1,31^ for lag times much smaller than the rotational time scale and when the direction of self-propulsion is parallel along the wall and height fluctuations are small.

### Parallel swimming assumption

To test if this requirement is fulfilled, we use a microscopy setup where the stage and the objective can be tilted (see ESI,† for more details). We record the trajectories of our active PS-swimmers in 0.1 wt% H_2_O_2_ at different tilt angles *θ* and track the particle positions using trackpy.^32^ We then calculate the MSD and fit it with eqn (1) up to lag times much smaller than the rotational timescale (0.5 s ≪ 70 s). We observe that *D* remains constant with *θ*, *cf.* ESI,† which implies that the particles swim at a constant distance from the substrate, irrespective of the tilt angle.

This also suggests a parallel orientation of the swimmers activity vector with respect to the substrate. That is, a coupling between the substrate and the swimmer orientation overcomes any gravitational torque from the heavier metal cap. Because swimmers of different speeds have the same diffusion constant (Fig. 4) and thus swim at the same height, we can assume that the swimmers propulsion force is aligned parallel to the substrate. If this would not be the case, a change in propulsion force, *i.e.* swimming speed, would change the force component directed perpendicular to the substrate. This would cause a change in height and hence in the diffusion constant. Since we do not observe this within measurement error, the assumption of parallel swimming holds. Therefore, in general we can compare the MSDs of different particles, as well as 2D and 3D experiments.

### 3D MSD analysis

To analyze our bulk measurements, we need to take into account the contribution from sedimentation to isolate the effects from the activity. We therefore apply a *z*-correction on the obtained 3D-trajectories by *z⃑*_cor._ = *z⃑*(*t*) − *t⃑v*_sedim_ and thus obtain the *z*-coordinate relative to a particle sedimenting at *v⃑*_sedim_. For *v⃑*_sedim_, we find that all passive particles sediment with a similar speed of |*v⃑*_sedim_| = 0.7 ± 0.1 μm s^−1^. This speed we extract from a total of 36 *z*-trajectories obtained using 9 passive particles by employing a linear fit, see ESI,† for details. After taking into account sedimentation, we can fit the MSD for swimmers far away from the substrate. For lag times *τ* smaller than the rotational timescale, we can describe the motion of the particle in 3D as

2 〈*r*^2^〉_3D_ = 6*Dτ* + *v*^2^*τ*^2^,

while *D* and *v* again are fit constants corresponding to the diffusion coefficient and speed. For both 2D and 3D experiments, lag times up to 1 s are fitted which is far below the rotational timescale.

### Height-dependent MSD analysis

**Fig. 3:**
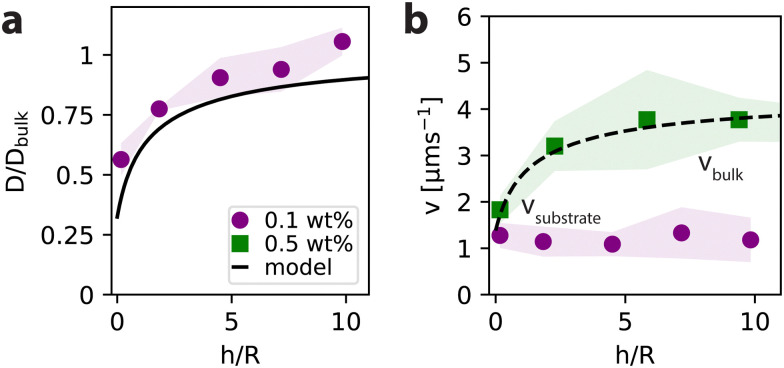
Height dependence of the diffusion coefficient and particle velocity. (a) For a single particle suspended in 0.1 wt% H_2_O_2_, the diffusion coefficient *D* normalized over the expectation value for *D*_bulk_ depends on the particle height above the substrate *h* normalized over the particle radius *R*. The evolution of *D*(*h*) over *D*_bulk_ follows the hydrodynamic model for the height-dependent diffusion derived by Faxén.^34^ (b) The single particle velocity *v*, however, is independent of the distance between particle and substrate for low activity (0.1 wt% H_2_O_2_), but becomes height-sensitive at higher activity (0.5 wt% H_2_O_2_), when the particle is closer than approx. 5 particle radii above the substrate. The dotted line in (b) serves to guide the eye. Shaded regions in all plots indicate the standard deviation between different particles under the same conditions. All shown experiments are in absence of salt.

Before measuring and comparing particle speeds at the substrate and in bulk, we first use the three-dimensional trajectories to determine the height at which the particles can be considered to move undisturbed in the bulk of the solution. To access this information, we divide the trajectory of a single particle into bins according to its distance from the substrate and calculate the MSD for every bin. The *z*-coordinate of the substrate is set equal to the initial *z*-value, as all trajectories started with the particle on the substrate. Over a total trajectory during which the acoustic tweezers were switched on multiple times, the *z*-coordinate of the substrate was noticed to change slightly. Therefore, we chose the first bin, which includes trajectories close to the substrate, to range from 0.75 μm above to 0.75 μm below the initial substrate height. In this bin, the data is treated as essentially 2D and the MSD is fitted with eqn (1). For all other bins, the data is treated as 3D and a bin size of 6 μm for experiments in 0.1 wt% H_2_O_2_ and 8 μm in 0.5 wt% H_2_O_2_ was chosen, which balances a higher resolution in height (number of bins) with good statistics in each bin (bin size). The MSD is then fitted with eqn (2) which corresponds to particles diffusing in 3D to obtain the fit constants *D* and *v*, see Fig. 3. Frames during the lifting of the particle with the acoustic tweezers and 1 second before and after that are not used in our calculations. Furthermore, only sufficiently long trajectories where the fit values *D* and *v* have a standard deviation smaller than 10% are used (see ESI,† for fits). All MSDs for the height-dependent data are fitted up to a lag time of 0.3 s. Finally, we normalize the diffusion constant with the one expected in the bulk as obtained from the Stokes–Einstein relation *D*_Stokes–Einstein_ = (*k*_B_*T*)/(6π*ηr*), where *T* = 296 K, *η* = 0.9321 mPa s (water),^33^ and *r* = 2.25 μm is used.

**Fig. 4:**
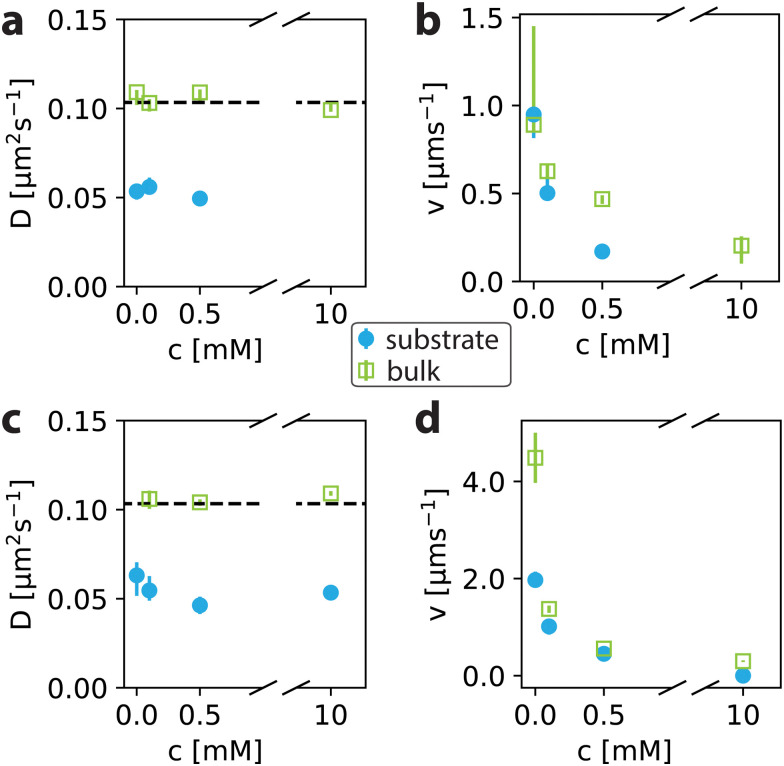
Salt affects active particles close to the surface and in bulk in a similar way. Diffusion coefficient *D* and particle velocity *v* both as a function of salt concentration for particles suspended in 0.1 wt% H_2_O_2_ ((a) and (b)) and 0.5 wt% H_2_O_2_ ((c) and (d)), respectively. Data obtained close to the substrate is indicated by blue spheres, and bulk data is indicated by green squares. *D* and *v* were obtained from a mean-squared displacement (MSD) analysis where lag times up to 1 s were considered. The dashed line in *a* and *c* indicates the expectation value from the Stokes–Einstein relation with *T* = 296 K and *η* = 0.9321 mPa s (water).^33^ Points plotted are median values and error bars indicate the first and third quartiles.

### Diffusion profile

The evolution of *D*(*h*) normalized over the bulk expectation value for *D*_bulk_ is shown in Fig. 3a. We find that starting from the surface where *h*/*R* ≈ 0, *D*(*h*)/*D*_bulk_ first sharply increases, before flattening out and approaching the bulk value. Despite being an active particle, the normalized values for the diffusion coefficient of the particle follow the hydrodynamic model derived by Faxén^34^ that reads *D*(*h*)/*D*_bulk_ = 1 − (9/16)*γ* + (1/8)*γ*^3^ − (45/256)*γ*^4^ − (1/16)*γ*^5^ with *γ* = *r*/(*h* + *r*). This relation was developed to capture the height-dependent diffusion *D*(*h*) of a passive, spherical particle above a no-slip wall, but similarly applies to the diffusion coefficient as extracted from the MSD of an active particle. Our results therefore confirm that it is possible to extract the particle-wall separation distance from the MSD of active particles, as was previously introduced by Ketzetzi *et al.*^24^

At 0.5 wt% H_2_O_2_, activity dominated already for small lag times, which made accurate extraction of the diffusive contribution difficult. That is, if the particle moves at a speed of 4 μm s^−1^ and has a diffusion constant of 0.1 μm^2^ s^−1^, at a frame rate of 33 fps, the activity term *v*^2^*τ*^2^ is of the same order as the diffusive term 6*Dτ* at the smallest lag time of 0.03 s, *i.e.*, 0.014 μm^2^ s^2^ and 0.018 μm^2^ s^2^, respectively. For small lag times, a diffusion constant can be obtained, see ESI,† however, these datapoints are sensitive to noise which results in the earlier described positive offset of the MSD at small lag times. Therefore, for the bulk experiment at 0.5 wt% H_2_O_2_ without salt, the diffusion constant is not fitted, but instead set to *D*_Stokes–Einstein_.

### Velocity profile

For *v*(*h*), we first look at the particles with a higher activity, *i.e.* those suspended in 0.5 wt% H_2_O_2_ (see Fig. 3b). The velocity first increases starting from *ca.* 2 μm s^−1^ close to the substrate before reaching a constant value of *ca.* 3.5 μm s^−1^ at heights corresponding to approximately 5 particle radii. However, when suspended in a lower fuel concentration of 0.1 wt% H_2_O_2_ (see Fig. 3b), the particles propel at a nearly constant velocity of about 1 μm s^−1^ at all heights. We conclude that the diffusion coefficient as well as the particle velocity at higher activity are sensitive to hydrodynamic effects which increase as the particle moves closer to the wall. This hydrodynamic influence of the substrate needs to be taken into account when comparing near-substrate and bulk behaviour of the active particles. Based on these observations, we define the bulk regime as starting from 5 particle radii above the substrate. Henceforth, bulk values for *D* and *v* are obtained from MSDs (of parts of the trajectories) at least 5*R* away from the substrate, see ESI,† for details.

### Effect of salt addition

Having established the range above which we can consider our measurements to be in bulk, we now examine how salt affects the behaviour of active particles in this regime. The diffusion constants and velocities resulting from the analysis of 〈*r*^2^〉_2D_ and 〈*r*^2^〉_3D_ for experiments done in 0.1 wt% H_2_O_2_ are shown in Fig. 4a and b. For all salt concentrations, the diffusion constant obtained for swimmers far away from the substrate matches the value from the Stokes–Einstein relation to within the standard error of the mean. *D*_Stokes–Einstein_ = (*k*_B_*T*)/(6π*ηr*) resulting in 0.103 μm^2^ s^−1^ for particles with *r* = 2.25 μm, *T* = 296 K and *η* = 0.9321 mPa s (ref. 33) (water) as indicated by the black dashed line in Fig. 4a. Close to the surface, the diffusion constant decreases to approximately half of the bulk value. Using an interpolation between two models^34,35^ for the height-dependent diffusion coefficient as was done in ref. 24, we find that this value corresponds to swimming heights of 0.38 μm. The relative diffusion constant *D*/*D*_bulk_ is in line with earlier observations.^24^ The decrease upon approaching the surface can predominantly be attributed to increased friction resulting from hydrodynamics.

Interestingly, we observe with increasing salt concentration that the velocities both close to the substrate and in bulk show a drastic decrease (see Fig. 4b). Already for 0.1 mM NaCl the velocities decrease significantly and drop further to being close to zero at 10 mM in 0.1 wt% H_2_O_2_. While this decrease is in line with previous measurements of catalytically active particles on substrates,^12,24^ in bulk this significant reduction has not been observed before. The decrease of velocity in bulk suggests that ionic species affect the particle activity that originates from particle surface effects and the presence of a wall is thus not necessary to observe this effect. The average velocity near the substrate seems to be a bit lower than the one in bulk, except for *c*_NaCl_ = 0 mM where the errorbar is larger. For 10 mM NaCl, insufficient data could be collected due to active particles sticking to the substrate which is why this data point was excluded in Fig. 4a and b.

Next, we investigate if the aforementioned velocity differences are enhanced at higher particle speeds, that is for a system with 0.5 wt% H_2_O_2_. At this higher fuel concentration, the diffusion constant for bulk-swimmers still matches the value expected from the Stokes–Einstein relation (dashed line, see Fig. 4c) and again decreases close to the wall due to hydrodynamics. Note that the bulk value of *D* for *c*_NaCl_ = 0 mM is not shown because of the dominance of the velocity term for particles moving at these speeds, as explained earlier. The averaged diffusion constants close to the wall obtained for experiments in different fuel and salt concentrations up to 0.5 mM are very similar, being (0.054 ± 0.007) μm^2^ s^−1^ and (0.058 ± 0.023) μm^2^ s^−1^ in 0.1 wt% H_2_O_2_ and 0.5 wt% H_2_O_2_, respectively. These small errors further support the assumption of a parallel activity vector with the substrate, at least to good approximation within the measurement error.

For swimmers at higher H_2_O_2_ concentration, we measure higher velocities. In the presence of salt (*c*_NaCl_ > 0 mM) the velocity decreases and particles move at similar speeds in bulk and close to the substrate. However, a notably large velocity difference is observed between bulk and near-surface swimmers in the absence of salt (0 mM NaCl) (see Fig. 4d). This difference could imply a surface effect that vanishes upon adding salt. However, at lower activity the difference in particle speed between bulk and substrate is within the error in the absence of salt (*c*_NaCl_ = 0 in panel b). If there would be a surface effect that vanishes upon adding salt, it seems to be absent at low activities.

Another explanation for the observed speed difference could be hydrodynamic effects. We base this insight on Fig. 3b, where the velocity profile seems to follow the same scaling as the hydrodynamic Faxén model, as indicated by the dashed line. If the swimmer speed follows a scaling relation with height, the absolute difference between substrate and bulk should be most pronounced at higher activity, since experimental noise might overrule small differences at lower activity. In Fig. 4b and d, higher fuel concentration and lower ionic concentration leads to faster particles, as well as a larger absolute difference in speed between substrate and bulk, which suggests coupling between the wall and the swimmer's speed. Notably, the coupling between swimmer and wall already strongly impacts the particle orientation and height, well before it affects the speed.

### Implications for the propulsion mechanism

**Fig. 5:**
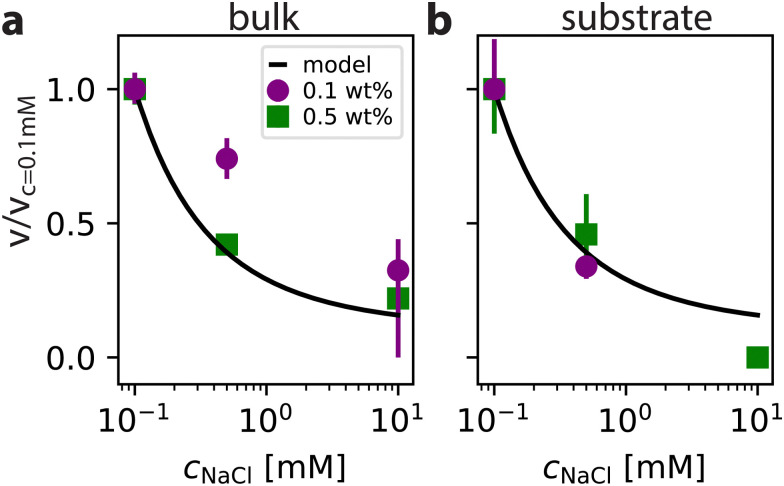
Relative speed reduction comparison. The velocities relative to the velocity at *c*_NaCl_ = 0.1 mM as function of *c*_NaCl_ in bulk (a) and at the substrate (b) for two different fuel concentrations compared with the effective Henry's function for self-electrophoresis *F*_H_(*κa*) (see footnote) following an electrokinetic theory outlined in ref. 25. Plotted points are median values and errorbars represent first and third quartiles.

We have seen that the effect of salt on the speed is equally present in the bulk as it is on the surface. This is a necessary step to gain a better understanding of the self-propulsion mechanism. The observation of a reduced swim velocity is consistent with the electrokinetic theory laid out in ref. 12 and 25. The salt concentration used in the experiment spans two orders of magnitude, *i.e.* we have *κa* ranging from ≈2 to 20, where *a* is the radius and *κ* the inverse (Debye) screening length¶¶For the purposes of this paper, we define *κ*^2^ = 2*e*^2^*c*/(ε*k*_B_*T*) with *e* the elementary charge, *ε* the dielectric constant of the medium, *k*_B_ Boltzmann's constant, and *T* the temperature. In our calculations we assumed *ε* ≈ 80*ε*_0_ with *ε*_0_ the vacuum permittivity and a temperature of *T* ≈ 300 K.. This lies outside the power-law scaling regime of the effective Henry's function for self-electrophoresis, *F*_H_(*κa*)||||The effective Henry's function for self-electrophoresis is given by 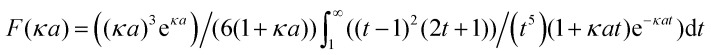 following ref. 25., as defined in ref. 25. This function governs how the self-propulsion speed of a particle decreases as a function of decreasing *κa*, when the only effect of salt on the particle speed is reducing the Debye screening length. Taking the speed at *c*_NaCl_ = 0.1 mM as a reference point, we predict a speed decrease by a factor of ≈0.4 by increasing the salt concentration to *c*_NaCl_ = 0.5 mM, and a decrease by a factor of ≈0.15 by increasing to *c*_NaCl_ = 10 mM, as shown in Fig. 5. This appears to hold in bulk for higher activity 0.5 wt% H_2_O_2_ with 0.41 and 0.22 for the speed ratios at *c*_NaCl_ values of respectively 0.5 mM and 10 mM relative to 0.1 mM, suggesting that our data aligns with the theoretical prediction, as shown in Fig. 5a.

However, the agreement is less good when looking at the bulk data for 0.1 wt% H_2_O_2_, where these speed ratios are 0.75 and 0.32 and the data could fit more closely a logarithmic trend. This mismatch may imply that salt has a secondary effect at low fuel concentration, or another mechanism is at play. Looking at the substrate data shown in Fig. 5b, the points at *c*_NaCl_ = 0.5 mM for both fuel concentrations match quite well with the theory, with 0.34 and 0.44 for fuel concentrations of 0.1 wt% and 0.5 wt%, respectively. The datapoint at *c*_NaCl_ = 10 mM is missing for the 0.1 wt% experiment as mentioned earlier. For the 0.5 wt% experiment, the activity is so low that the obtained speed from fitting is equal to zero. This makes it hard to assess the quality of the trend. However, what is noticeable is that the trend for 0.1 wt% substrate data strongly departs from that found in bulk, suggesting that the propulsion mechanism might have a different dominant component under these conditions. This difference in trend could also be due to the coupling between the substrate and particle being affected by salt, similar to osmotic counterflows as proposed in earlier work.^24^ Additional experiments will need to be conducted before conclusions can be drawn about the specific self-propulsion mechanism (or mechanisms) that is (or are) at play in our catalytic self-propelled particles.

In summary, we have successfully quantified the diffusion constant and speed of active particles as a function of the distance from a wall. From this measurement, we could define the bulk behavior as starting five particle radii away from the substrate. We used this to measure the speed of active particles in bulk and compared it to particles moving close to a wall. Our results show that salt lowers the particle speeds both in 2D and 3D. At low activity, the measured particle speeds at different salt concentrations in both cases are very similar which suggests that ionic wall-effects have little effect on the particle speed. However, at higher activity we observe a speed difference between substrate and bulk that gets bigger with faster moving particles. This points towards an activity-dependent wall-effect of which the velocity height profile follows a scaling similar to a hydrodynamic model.

Furthermore, the observed speed reduction with increasing ionic concentration follows a scaling predicted by an electrokinetic theory for both microswimmers near a substrate and particles with a high activity in bulk. Particles with a lower activity in bulk follow a different scaling, which suggests a different propulsion mechanism, but additional experiments are needed. Our experiments thus not only provide a better understanding of ionic effects on microswimmers in 3D, but also suggest a coupling between the wall and the swimming speed.

## Author contributions

Julio Melio: conceptualization, methodology, formal analysis, investigation, writing – original draft, visualization. Solenn Riedel: conceptualization, methodology, investigation, writing – original draft, visualization. Ali Azadbakht: conceptualization, methodology, investigation. Silvana Caipa Cure: methodology, investigation. Tom M. J. Evers: investigation. Mehrad Babaei: investigation. Alireza Mashaghi: resources. Joost de Graaf: conceptualization, methodology, formal analysis, writing – original draft, funding acquisition. Daniela J. Kraft: conceptualization, methodology, resources, writing – original draft, supervision, funding acquisition.

## Data availability

Data for this article, including the particle traces of all experiments are available at 4TU.ResearchData at https://doi.org/10.4121/0a058f2f-09ce-4637-a22f-d30789c35ccd. Analysis files supporting this article have been included as part of the ESI.†

## Conflicts of interest

There are no conflicts to declare.

## Supplementary Material

SM-021-D4SM01387E-s001

SM-021-D4SM01387E-s002
