# Supplementary material for: The motion of catalytically active colloids approaching a surface
Source: Soft Matter. 2025 Mar 5;21(13):2541–7. doi: 10.1039/d4sm01387e (PMC11894793; doi:10.1039/d4sm01387e)
Supplement: SM-021-D4SM01387E-s001 [file SM-021-D4SM01387E-s001.pdf]

Supplementary Information (SI) for Soft Matter.
This journal is © The Royal Society of Chemistry 2025

Supplementary Information: The motion of catalytically active colloids approaching a surface

Julio Melio,[1] Solenn Riedel,[1] Ali Azadbakht,[1] Silvana A. Caipa Cure,[2] Tom M.J. Evers,[2] Mehrad Babaei,[2] Alireza Mashaghi,[2] Joost de Graaf,[3] and Daniela J. Kraft[1]

[1]*Huygens-Kamerlingh Onnes Laboratory, Leiden University, P.O. Box 9504, 2300 RA Leiden, The Netherlands*
[2]*Medical Systems Biophysics and Bioengineering, Leiden Academic Centre for Drug Research, Faculty of Science, Leiden University, 2333CC, Leiden, The Netherlands*
[3]*Institute for Theoretical Physics, Center for Extreme Matter and Emergent Phenomena, Utrecht University, Princetonplein 5, 3584 CC, Utrecht, The Netherlands*
(Dated: February 14, 2025)

I. TILTED STAGE MICROSCOPY

The tilted stage microscopy setup used to determine the diffusion constant at different tilt angles is shown in Fig. S1a. This setup consist of an inverted microscope (Nikon Ti-E) equipped with an arm (LSMTech InverterScope®) onto which a 10× air objective was mounted. The arm is able to rotate the objective as can be seen in Fig. S1b. Next to the microscope, a stage that could move in x and y and rotate with the objective was assembled using two linear stages and an angle plate. A 660 nm LED and a condensor lens were used to illuminate the sample. A diffuser was used to improve image quality (not shown).

Results of the diffusion constants at different tilt angles from MSD analysis are shown in Fig. S1c. The diffusion constant stays constant (within error) which suggests that the particles keep swimming at the same distance from the substrate, regardless of the tilt angle.

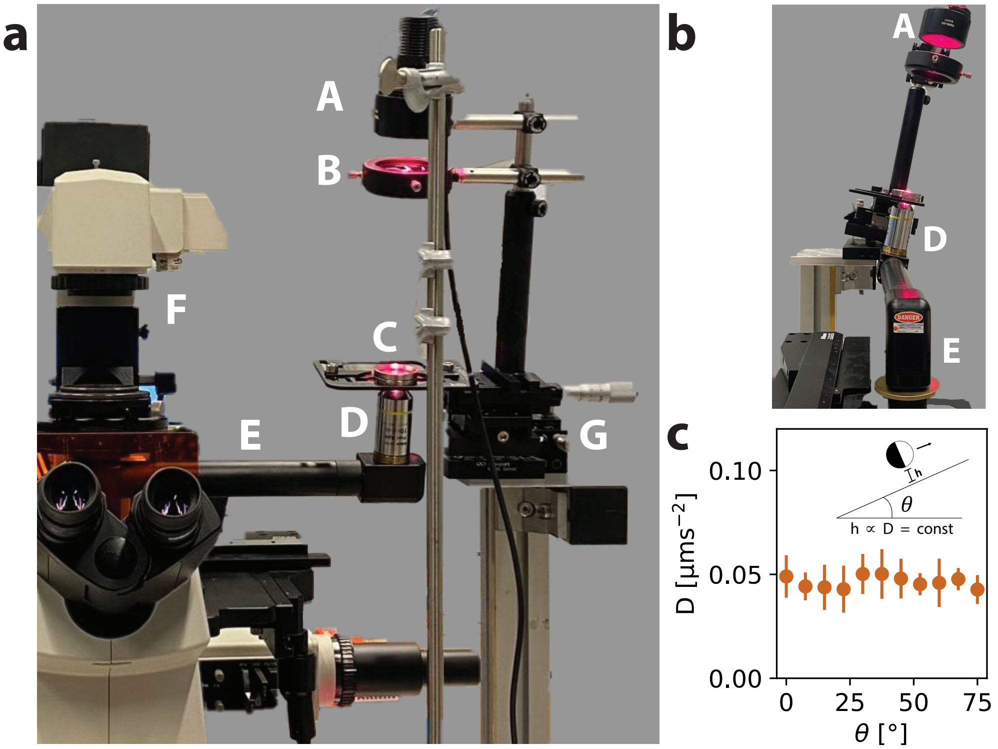

FIG. S1. *Tilted stage setup.* (a) Experimental setup where the stage and the objective of the microscope can be tilted. The microscope (F) is equipped with a rotating arm (E) on which a 10× objective (D) is mounted. The sample holder is placed on a stage that can be moved in x and y with the help of 2 linear stages and tilted to align with the objective using an angle plate (C and G). A 660 nm LED (A) and condensor (B) illuminate the sample. (b) Side view of the tilted setup. (c) Diffusion coefficient D at different tilt angles θ resulting from the MSD analysis.

II. SEDIMENTATION SPEED

The sedimentation speed was determined by analyzing in total 36 sedimentation occurrences of 9 different particles. The particles are the same as used in all experiments (4.5 μm PS, 5 nm Pt/Pd) and these sedimentation experiments are done in water, which prevents active propulsion. In Fig. S2, the sedimentation trajectories are shown. For all trajectories, the first z-coordinate z_0 has been subtracted to make all trajectories start in the same point. The resulting data was then fitted with a linear function $z = at$, where a is the fit constant corresponding to the sedimentation speed. From the fit, $a = -0.7 \pm 0.1$ μms^{-1} was extracted.

For some trajectories, large displacements in z between consecutive frames were observed for a specific height, which seems to indicate an inconsistency in the LUT of that particle. This could result in a positive offset of the MSD, reducing the quality of the fit. MSDs with an offset are therefore filtered out by only selecting MSDs where the difference between the first point, smallest lag time, and the fit is smaller than 50% of the fit.

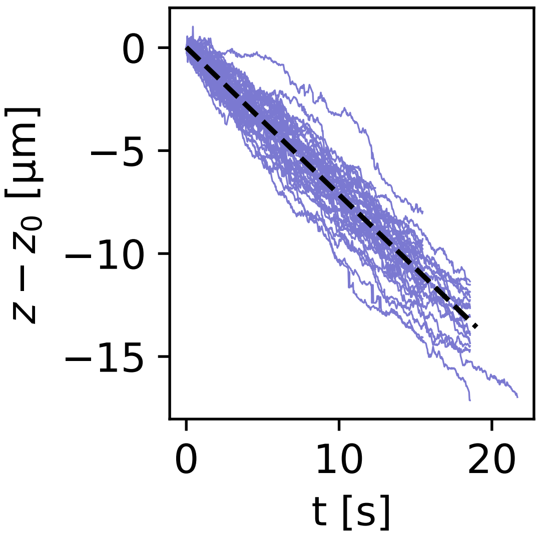

FIG. S2. *Sedimentation speed.* z-coordinate of trajectories of passive particles (PS-Pt/Pd particles in water) and the linear fit indicated with the black dashed line. The fit yields an average sedimentation speed of 0.7 ± 0.1 μms^{-1}.

III. ADDITIONAL INFORMATION ON MEAN-SQUARED DISPLACEMENT ANALYSIS

A. Height dependent mean squared displacements

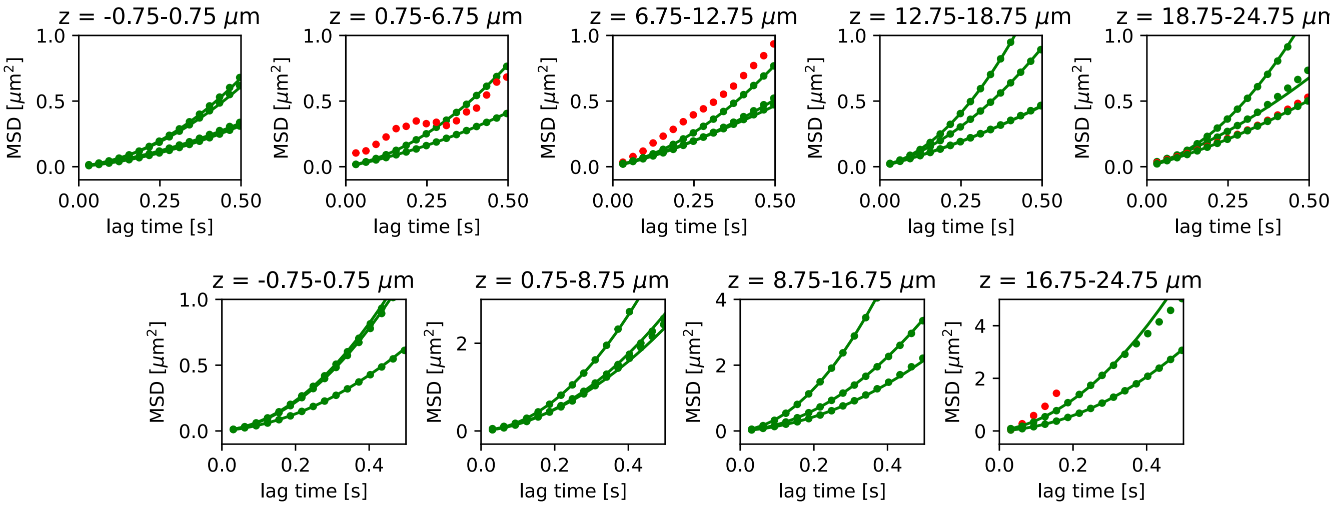

FIG. S3. *Height-dependent MSDs.* The Mean Squared Displacements (MSDs) for parts of the trajectory that fall into different height bins with respect to the substrate. The height bin ranges are indicated in the titles of the subplots. The upper graphs are for experiments in 0.1 wt% H_2O_2 and the lower graphs are for experiments in 0.5 wt% H_2O_2, both in absence of salt. MSDs that are sufficiently long, i.e. where the fitted D and v have a standard error smaller than 10%, are shown in green, while the other MSDs are shown in red.

B. Fit quality and variance across sample

In Fig. S4, the individual MSDs are shown with their corresponding fits. The diffusion constants and velocities reported in the main text were extracted from this data. Both the good quality of the fit as well as the variance between particles can be observed in this representation.

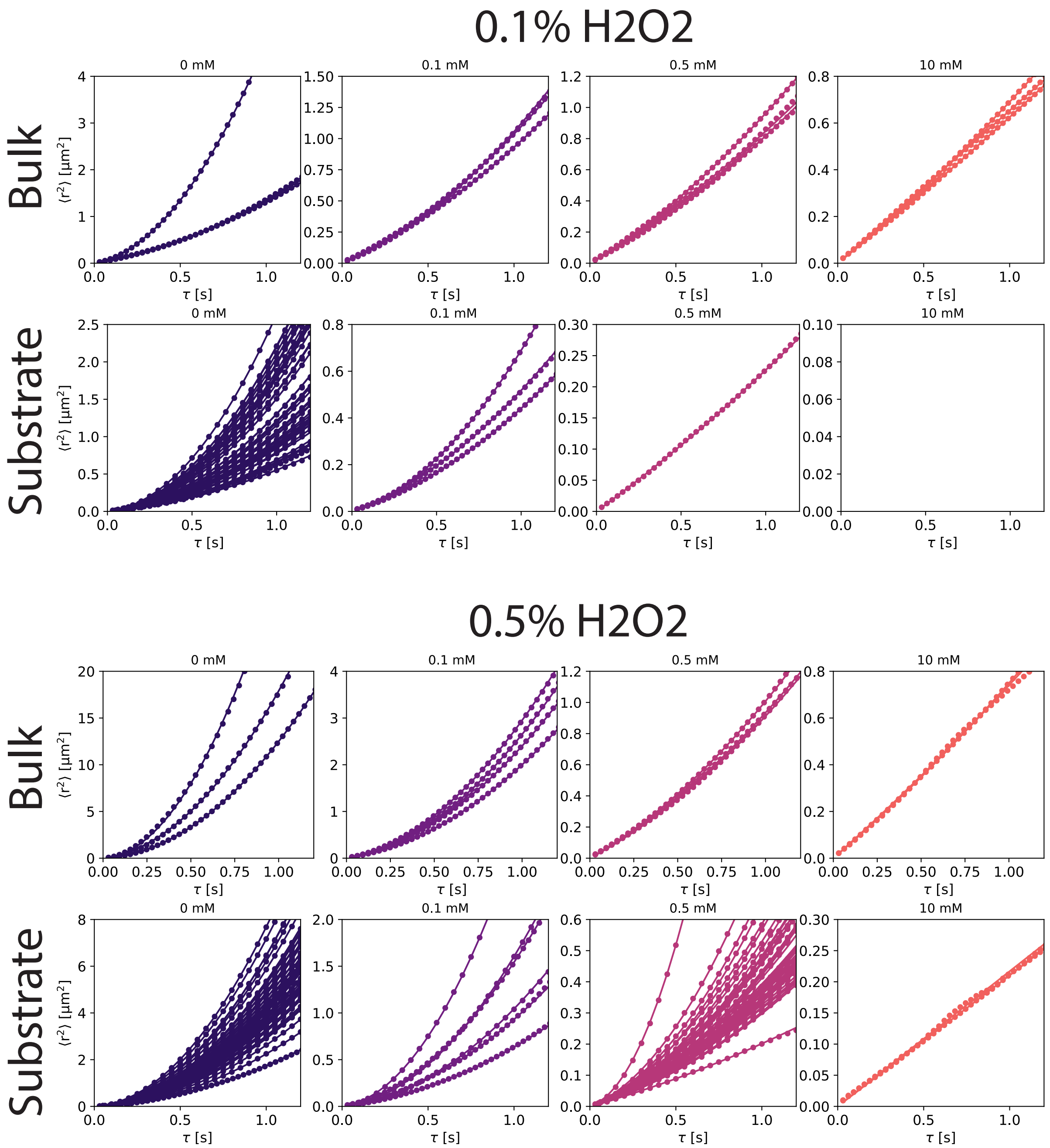

FIG. S4. *Mean-squared displacements and their fits.* For experiments in bulk and on a substrate, the measured MSDs from the trajectories are shown (indicated with points) and the fits are shown (indicated with a solid line). Lag times up to 1 s were considered for the fit.

C. Temporary motion at certain height

Some z-trajectories contained large displacements at specific heights, of which an example is shown in Fig. S5. The displacement distribution in the marked region is plotted on the right and shows two small peaks at low and high Δz. Because this is the displacement between consecutive frames, this will create an offset in the MSD. Therefore, only MSDs are selected where the difference between the fitted line and the first point, corresponding to the smallest lag time, is smaller than 50% of the value of the fitted line.

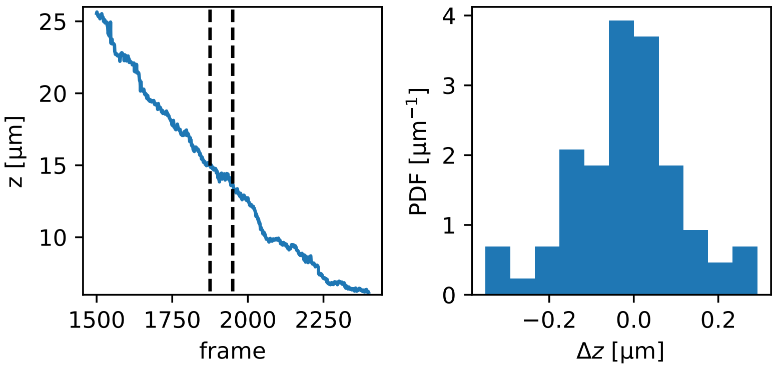

FIG. S5. *Larger displacements in z.* An example of a particle's z trajectory where large displacements are observed at a specific height, leading to the shown displacement distribution that has small peaks around $\pm$ 0.3 μm.

D. MSDs for different speeds and diffusion constants

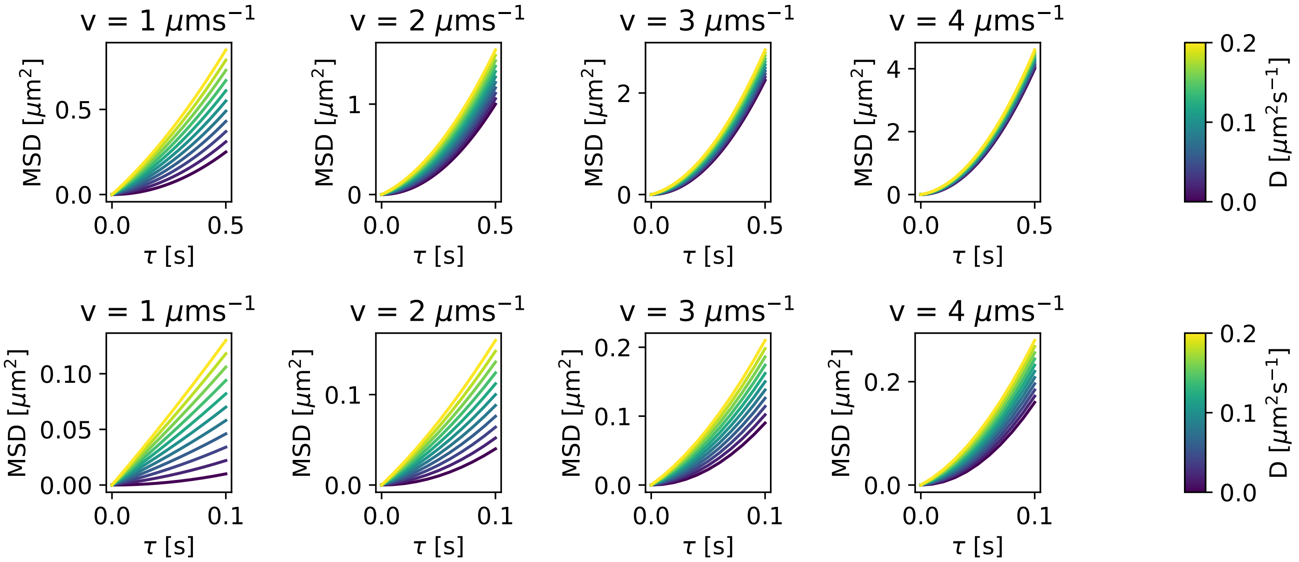

FIG. S6. *MSD for different speeds and diffusion constants* The mean squared displacement as calculated from $6D\tau + v^2\tau^2$ is plotted for different values of D (represented by color) and different values of v (indicated in the title) up to a lag time of $\tau = 0.5$ s (top row) and $\tau = 0.1$ s (bottom row).
